# Efficacy and cost‐effectiveness of extended nursing roles in dementia care: Results of the cluster‐randomized trial InDePendent

**DOI:** 10.1002/alz.70727

**Published:** 2025-10-27

**Authors:** Anika Rädke, Bernhard Michalowsky, Fabian Kleinke, Moritz Platen, Annelie Scharf, Michelle Pfaff, Maresa Buchholz, Franka Mühlichen, Peter Penndorf, Stefanie Schade, Janina Dombrowski, Pia Elisa Madaida Lerch, Katrin Christiane Reber, Manon Austenat‐Wied, Cornelia Martens, Neeltje van den Berg, Wolfgang Hoffmann

**Affiliations:** ^1^ German Center for Neurodegenerative Diseases (DZNE) Site Rostock/Greifswald Greifswald Germany; ^2^ Institute for Community Medicine University Medicine Greifswald Greifswald Germany; ^3^ AOK Nordost ‐ Die Gesundheitskasse Versorgungsprogramme und Verträge Potsdam Brandenburg Germany; ^4^ Techniker Krankenkasse Landesvertretung Mecklenburg‐Vorpommern Schwerin Mecklenburg‐Vorpommern Germany

**Keywords:** advanced nursing, cost‐effectiveness, dementia, primary care, randomized controlled trial, unmet needs

## Abstract

**INTRODUCTION:**

Extended nursing roles in dementia can improve treatment, care, and patient and caregiver outcomes. We tested the efficacy and cost‐effectiveness in people living with dementia (PlwD) and caregivers.

**METHODS:**

We analyzed data from 417 PlwD within the multicenter, cluster‐randomized InDePendent trial six months after baseline. Specifically, qualified nurses carried out advanced dementia care management individually tailored to each patient's and caregiver's needs. Outcomes included unmet needs (CANE), quality of life (QoL‐AD, EQ‐5D), caregiver burden (Zarit), and cost‐effectiveness (FIMA, RUD).

**RESULTS:**

PlwD receiving the intervention had 74% lower unmet needs (0.26, CI^95%^: 0.17 to 0.40, *p* < 0.001) and higher quality of life (0.04, CI^95%^: 0.01 to 0.07, *p* = 0.017) than controls. There was a gain in quality‐adjusted life years (0.01, CI^95%^: −0.001 to 0.018) at slightly higher costs (1,425 EUR, CI^95%^: 638 to 2,211). There was no effect on caregiver burden after six months.

**DISCUSSION:**

The results provide evidence for the efficacy and cost‐effectiveness of extended nursing roles in dementia care, demonstrating that extended nursing roles reduce unmet needs and improve quality of life.

**TRIAL REGISTRATION:**

This trial was prospectively registered on ClinicalTrials.gov under the identifier NCT04741932 on February 2, 2021.

**Highlights:**

- The InDePendent intervention significantly reduced the number of unmet needs in PlwD and their caregiver according to CANE.
- Extended nursing roles can improve PlwD's health‐related Quality of Life.
- Advanced dementia care management were likely to be cost‐effective after six months.

## 1 BACKGROUND

People living with dementia (PlwD) often suffer from numerous comorbidities in addition to their cognitive impairment, causing a variety of unmet needs, such as a timely diagnosis, and require interdisciplinary and individualized post‐diagnostic support.^1^, ^2^, ^3^ However, PlwD face substantial barriers in accessing care and support, which originate not only from individual‐level factors but also from broader structural gaps within the healthcare system. These include limited awareness and information, stigma, inadequate tailoring of services to needs, and sociodemographic challenges such as higher age and living situation. In particular, there is no key contact person to connect PlwD with healthcare services and support programs alongside an individual care pathway to identify and address existing unmet needs.^4^ Dementia care programs attempt to address these challenges by providing advocacy, structured guidance, and improved care navigation.

Thus, new healthcare models are needed to ensure individualized, high‐quality medical and nursing care for PlwD.^5^ A redistribution of responsibilities within primary care, often referred to as “extended nursing roles,” could be one approach to addressing the need for innovative models and care programs.

Internationally, such roles have been formalized through the development of advanced practice nursing, in which graduate‐educated nurses with advanced competencies work collaboratively within healthcare teams and contribute to care delivery, including assessments, diagnostic reasoning, prescribing, and independent patient management.^6^, ^7^ In several countries, nurses with appropriate qualifications complement primary care by acting autonomously, thereby helping to expand care capacities and improve access to timely support.

By 2019, several European countries had implemented nurse prescribing laws supported by formal education and regulatory frameworks. These roles have demonstrated high adaptability, particularly during health crises such as the COVID‐19 pandemic, and have contributed to improved care quality and patient outcomes across various settings.^7^, ^8^ In contrast, the German nursing system has traditionally followed a vocational training model with a low proportion (3%) of academically trained nurses.^9^ The majority of nurses complete a 3‐year non‐academic training program and enter practice without a university degree. Although bachelor‐level nursing programs have been introduced in recent years, they are not yet the standard pathway.^10^ To address evolving care needs, several national initiatives have explored new competencies for nurses through structured training programs, resulting in extended nursing roles. These roles equip nurses – often through post‐vocational or bachelor‐level training – with skills in areas such as care coordination, needs assessment, and patient counseling. However, unlike nurse practitioner roles in countries such as the United States or United Kingdom, which may include independent prescribing rights, extended roles in Germany are currently legally limited to approved model projects within the statutory healthcare system (§63,3c and §64d Social Code Book V). These legal frameworks include specific predefined tasks in some selected disease areas (such as dementia) and allow piloting extended nursing roles in temporally restricted model projects.

Studies have revealed that extended nursing roles can yield comparable or superior patient and health system‐level outcomes.^11^, ^12^ Collaborative dementia care programs, often delivered by specially trained nurses, are typically based on the redistribution of tasks and the integration of extended nursing roles. These programs aim to improve post‐diagnostic support and coordinate treatment and care in close cooperation with the general practitioner and have been successfully implemented in several countries.^13^, ^14^, ^15^, ^16^ Collaborative dementia care management programs are defined as interventions that coordinate individualized, guideline‐based care for PlwD and their caregivers in primary care settings.^13^, ^15^ Delivered by qualified nurses, dementia care management interventions typically include structured needs assessments, medication management, caregiver support, interdisciplinary care planning, and care coordination. Meta‐analyses and previous randomized controlled trials (RCTs) have demonstrated that dementia care management programs can reduce neuropsychiatric symptoms, improve caregiver burden and health‐related quality of life (HRQoL), delay institutionalization, and increase anti‐dementia drug prescription rates.^14^, ^17^ However, evidence concerning the efficiency and cost‐effectiveness of extended nursing roles is limited and remains heterogeneous.

This analysis aims to evaluate the efficacy and cost‐effectiveness of a dementia care model with extended nursing roles in an outpatient setting of physician networks in Germany compared to usual care, focusing on patient, caregiver, and health system‐relevant outcomes.

## 2 METHODS

### 2.1 Trial design and participant flow

The InDePendent (Interprofessional Dementia Care: Redistribution of tasks between physicians and qualified nurses in primary care) trial is a multicenter, cluster‐randomized controlled intervention study with two arms (intervention group and waiting‐control group receiving usual care). The trial was designed to test the efficacy and cost‐effectiveness of advanced dementia care management with a redistribution of tasks between nurses and physicians in primary care.

The study protocol^18^ was approved by the ethics committees of the Chamber of Physicians of Mecklenburg–Western Pomerania and the State Medical Council Brandenburg and Hesse. The study was funded by the German Innovation Fund of the Federal Joint Committee (G‐BA) (ref.: 01NVF18034).

**RESEARCH IN CONTEXT**

1. **Systematic review**: The authors reviewed the literature using PubMed. Studies revealed that extended nursing roles could satisfy patients and cause similar or better patient and health system‐relevant outcomes. Several countries have implemented such care models to improve post‐diagnostic support and coordinate treatment and care, which generally supported previous studies evaluating extended nursing roles. However, evidence concerning the efficacy and cost‐effectiveness of extended nursing roles is rare and needs to be confirmed.
2. **Interpretation**: The findings of this study led to the hypothesis that an extension of nursing roles, in which nurses autonomously perform specific medical tasks traditionally carried out by physicians, would reduce the number of unmet needs of PlwD and their caregivers compared to routine care and would likely be cost‐effective by improving QALYs at slightly higher costs.
3. **Future directions**: Our conclusion highlights the issue of early initiation of post‐diagnostic support in dementia through extended nursing roles in primary care. Further research with larger samples, extended intervention and observational periods, and varying settings is needed to reveal (1) who benefits most from such approaches, (2) how such approaches could be modified and extended, and (3) where an implementation is most appropriate.

General practitioner and specialist (neurologists, psychiatrists) practices (*n* = 236) of five physician networks in three federal states of Germany (Mecklenburg–Western Pomerania, Hesse, and Brandenburg) were invited to participate. One hundred forty‐nine practices (63%) agreed (informed consent) to participate and were randomized (ratio 1:2) to the intervention (dementia care management with extended nursing roles) or waiting‐control group (usual care) using cluster‐level block randomization.^19^ Practices were the unit of randomization to reduce the risk of contamination across groups. Neither practices nor participants were informed about their randomization status but became aware of their status in the course of the study due to the nature of the intervention.

Physicians (general practitioners or specialists) systematically assessed the following eligibility criteria of patients: age ≥70 years, living community‐dwelling, formal dementia diagnosis, or positively screened for dementia (Dementia Detection Test (‘DemTect’) ≤ 8).^20^ Patients who fulfilled the criteria were informed about the study, invited to participate, and asked to provide written informed consent. If the patient named a caregiver, he or she was also asked to participate. When patients were unable to provide written informed consent, their legal representative was asked to sign the informed consent on their behalf.

**FIGURE 1:**
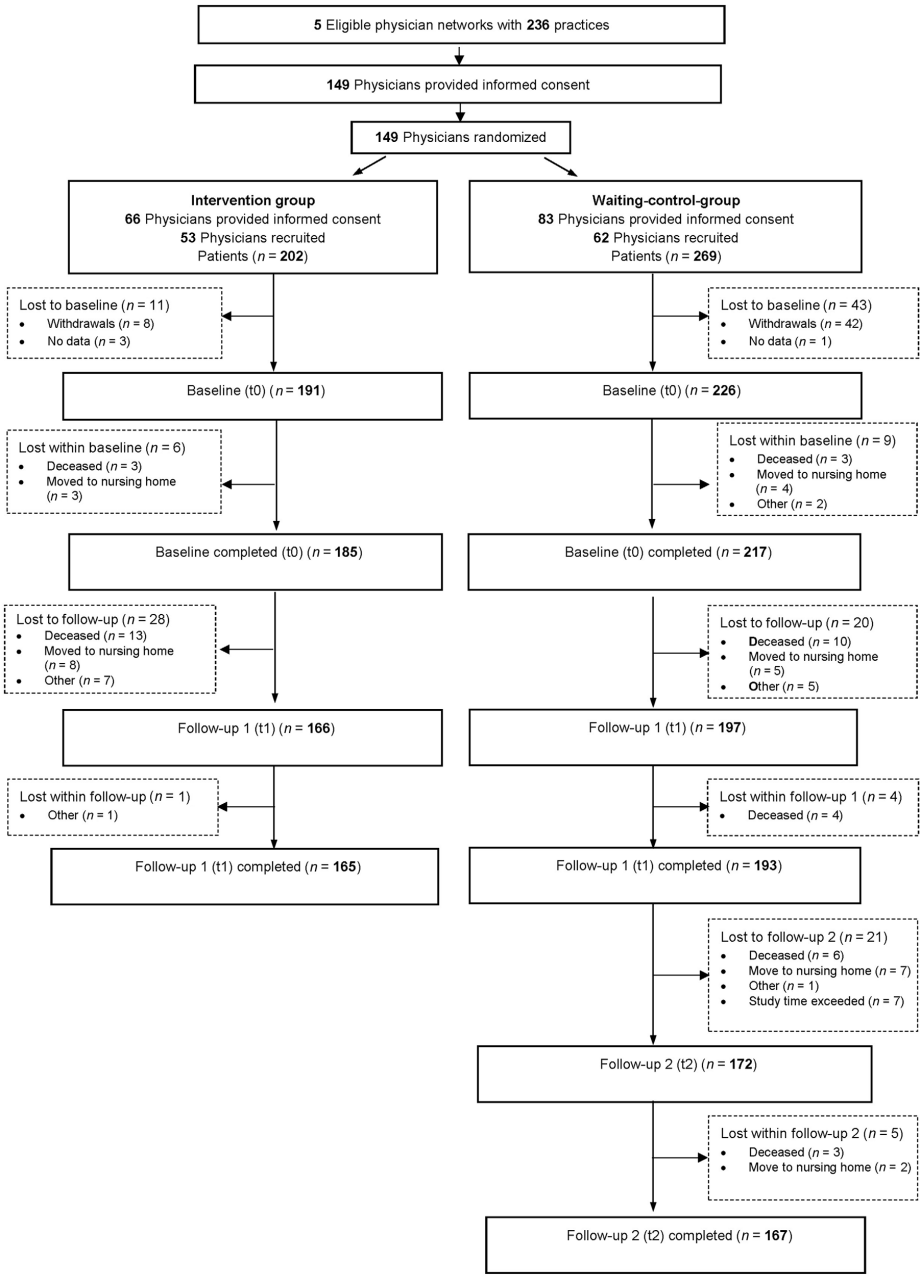
Trial flow chart.

Four hundred seventy‐one patients fulfilled the eligibility criteria and agreed to participate. Four hundred seventeen patients started the baseline assessment. Fifty participants withdrew their informed consent, and four suspended the 6‐month follow‐up assessment. Figure 1 and Table S1 display the trial flow chart and drop‐out information, indicating that the missing data are missing at random.

### 2.2 Dementia care management intervention with extended nursing roles

The intervention aims to optimize treatment and care of PlwD by dementia‐specific qualified nurses with expanded competencies. These nurses autonomously perform specific medical tasks traditionally carried out by physicians (e.g., prescribing medical and care aids and outpatient care services, issuing follow‐up prescriptions, documenting and reviewing medication regimens, providing counselling, administering infusion therapies and injections, applying standardized assessment tools, and initiating referrals and consultations with contracted physicians), within the framework of a model project paragraph in the Social Code Book V (Sektion 63, Paragraph 3c SGB V). The goal was to promote person‐centered, needs‐based, and coordinated care for PlwD.

The intervention was based on a collaborative Dementia Care Management model, previously implemented and evaluated in the DelpHi‐MV study.^17^ For the InDePendent trial, this model was adapted to incorporate an expanded nursing role and a structured redistribution of selected responsibilities between qualified nurses and general practitioners. Importantly, the aim of the expanded nursing tasks was not to substitute for physicians but rather to support and complement their work within an interdisciplinary care framework. While certain medical activities were performed autonomously by the nurses, all actions were systematically coordinated with the treating general practitioner. Regular communication and joint clinical decision‐making between nurses and physicians facilitated the integration of care processes and promoted continuity and quality of care across healthcare professions.

Prior to the start of the InDePendent intervention, participating nurses completed a structured qualification program to become certified Dementia Care Managers. The training was developed by the German Center for Neurodegenerative Diseases (DZNE) and approved by the German Federal Ministries of Health and Family Affairs. The program comprised 366 hours of theoretical instruction and 344 hours of practical training, delivered part‐time over 9 months via a web‐based three‐dimensional learning environment. The program included basic and advanced modules and practical internships covering dementia‐specific care, care planning, coordination, and selected medical tasks (e.g., re‐prescription of medical and care‐related aids like nursing care articles, showering aids, and mobility aids such as rollators, ordering diagnostics), which the specific qualified nursing can then carry out independently. Training was delivered by physicians and academically trained nurses and concluded with a formal state examination. Competency acquisition was monitored throughout the study via examinations and structured evaluations. The qualification enabled nurses to assume extended roles within primary dementia care in close collaboration with general practitioners.

Following qualification, the Dementia Care Managers implemented the intervention in participants’ homes, aiming to provide optimized care to reduce unmet care needs. The intervention, based on dementia‐specific guidelines,^21^, ^22^, ^23^ provided an individualized care scheme integrating multiprofessional and multimodal strategies to improve patient‐ and caregiver‐related outcomes.^24^ It comprised five parts: (1) comprehensive assessment of unmet medical, pharmaceutical, psychosocial, social, and nursing care needs; (2) development of an individualized treatment and care plan; (3) implementation of the plan to address unmet needs; (4) monitoring of the intervention; and (5) outcome assessment.

The assessment was carried out over one to four home visits to identify needs with the help of an algorithm‐based computerized Intervention Management System. This enabled the specialized nurses to swiftly and systematically identify needs, facilitate the development of the individualized care plan, and later on monitor the implementation of intervention tasks. Details on the design, the qualification, the intervention, and functionalities of the Intervention Management System are explained elsewhere.^24^, ^25^, ^26^, ^27^

Upon completion of the assessment, the nurses sent out brief summaries and recommendations to the treating general practitioner. Subsequently, the nurse and the physicians implemented the intervention tasks in close cooperation with various healthcare providers and other health and social care stakeholders. The intervention included at least monthly consultations with participating PlwD and caregivers over six months. Monthly case consultations with other care managers and nursing scientists were carried out as needed. Participants of the waiting‐control group received usual care for six months and subsequently the intervention.

### 2.3 Baseline data and outcome assessment

Standardized, computer‐assisted, face‐to‐face interviews were conducted between January 2021 and August 2023 at the participants' homes by study staff following study inclusion at baseline and six months after baseline. Analyses were based on the six‐month follow‐up data. Baseline characteristics of the trial have been described elsewhere.^28^ The reporting of this study follows the CONSORT statement^29^ and CHEERS guidelines.^30^

#### 2.3.1 Sociodemographic and clinical outcomes

Age, sex, living situation, and presence of an informal caregiver and the following clinical measures were assessed: cognitive impairment according to the Mini‐Mental State Examination (MMSE),^31^ functional impairment according to the Bayer‐Activities of Daily Living Scale (B‐ADL),^32^ mobility status by De Morton Mobility Index (DEMMI),^33^ and depression measures by the Geriatric Depression Scale (GDS).^34^ We examined all International Classification of Diseases, Tenth Revision diagnoses documented in the practitioner's records and reviewed all medications following the medication plan.

#### 2.3.2 Efficacy Outcomes

The primary outcome was the number of unmet needs assessed with the German version of the Camberwell Assessment of Need for the Elderly (CANE).^35^ CANE, a validated instrument that substantially contributes to a comprehensive assessment of needs in the elderly, includes 24 daily life domains (plus two caregiver items) to evaluate the physical, psychological, social, and environmental met and unmet needs of older people from both the patient's and caregiver's (proxy) perspectives, resulting in a total number of unmet needs ranging from 0 to 27.^36^ Minimal important differences (MIDs) estimates did not exist.

The secondary outcomes included

1. Quality of life, measured by the Quality of Life in Alzheimer's Disease (QoL‐AD),^37^ ranging from 13 to 52, in which higher numbers indicate higher quality of life, and the EQ‐5D‐5L,^38^ covering five dimensions. A health utility index was calculated using the German value set,^39^ with values ranging from 0 (worst) to 1 (best). Both measures are well validated. MIDs exist only for the EQ‐5D‐5L (non‐surgical interventions: 0.05).
2. Caregiver burden, measured by the validated Zarit Burden Inventory (ZBI),^40^ ranging from zero to 88 points, with higher scores representing higher levels of subjective caregiver burden.^41^ MIDs estimates did not exist.

#### 2.3.3 Cost‐effectiveness outcomes

General health status was measured by the EQ‐5D‐5L. Costs were calculated from a societal and public payer perspective based on unit costs. Utilization of healthcare services and informal care were assessed using the Questionnaire for Health‐Related Resource Use in an Elderly Population (FIMA)^42^ and the Resource Utilization in Dementia (RUD) questionnaire, covering caregiver support in hours per month for (i) Activities of Daily Living (ADL) (e.g., personal hygiene, eating, and dressing) and (ii) instrumental ADL (IADL) (e.g., shopping, meal preparation, and housekeeping).^43^

### 2.4 Statistical methods

#### 2.4.1 Descriptive statistics

Demographics and clinical variables were descriptively demonstrated across groups for sample description. Differences between study groups at baseline were assessed using generalized linear and logistic regression models with random effects for clusters (general practitioner practices and physician network).

#### 2.4.2 Efficacy analysis

We analyzed the intervention effect after six months for the primary study outcome, number of unmet needs, and the secondary outcomes, caregiver burden, and quality of life.

For missing data, we applied a multiple imputations technique by chained equation.^44^, ^45^ Prior to imputation, a drop‐out analysis was performed to evaluate the missing data mechanism. Using multivariate logistic regression with random effects for general practitioners, we examined whether dropout was associated with observed baseline variables, including study group, unmet needs, sex, availability of an informal caregiver, living situation, and age (Table S1). While no variables reached statistical significance, unmet needs and sex showed trends toward influencing dropout, suggesting that missingness was likely related to some observed variables. Consequently, the assumption of missing at random (MAR) was deemed appropriate, and the applied imputation method accounts for this.

The primary efficacy analysis was carried out using multivariable Poisson (CANE, count variable) and linear regression models (HRQoL and caregiver burden, metric variable), making it possible to adjust for relevant covariates (e.g., age, sex, living situation, baseline score). Models based on the intention‐to‐treat (ITT) principle (full analysis set, excluding withdrawing PlwD) to preserve benefits of randomization and provide unbiased estimates of the intervention effect six months after baseline compared to usual care under real‐world conditions.

A per‐protocol analysis (PPA) was performed as a sensitivity analysis for the primary outcome (number of unmet needs), including PlwD, who had completed the study according to the protocol. We defined the PPA as complete case analyses, including PlwD adhering to the intervention and assessment period of five to eight months. Participants with missing data in the assessments or study protocol violations (less than five months of intervention or follow‐up assessment or more than two months delayed) were excluded from the PPA.

In all analyses, the predictor of interest was the study group (dummy variable). For statistical comparison between groups, *t*‐tests (unadjusted means) and multivariate regression models adjusted for sex, age, living situation, and the respective baseline value of the dependent variables (adjusted means) to account for inter‐individual variance at baseline and to reduce residual variance were calculated. A positive intervention effect was defined as a statistically significant back‐transformed regression coefficient (for the primary outcome) or as a statistically significant adjusted mean difference (AMD) (for the secondary outcomes) between the study groups at *p* < 0.05. We also used random effects for the physician network and the respective physician practice to account for dependencies. All *p* values for the primary analyses are one‐sided. Statistical analyses were conducted using SPSS version 29.0., StataSE16.1 and RStudio.

#### 2.4.3 Cost‐effectiveness analyses

Costs were calculated from a societal and public payer perspective using 2023 unit costs.^46^, ^47^ The payer perspective (based on the total sample) was chosen as the primary analysis and the societal perspective (based on a subsample of patients with an informal caregiver) as sensitivity analysis. The health utilities obtained from the EQ‐5D‐5L scores at baseline and follow‐up were used for quality‐adjusted life years (QALYs) calculation.

The incremental cost‐effectiveness ratio (ICER) was calculated using the incremental cost per QALY gained by the intervention compared to usual care.^48^ Due to baseline value differences, incremental costs and QALYs were estimated using linear regression models adjusted for age, sex, living situation, baseline utility score, and baseline costs. To handle sampling uncertainty, non‐parametric bootstrapping with 2,000 resamples stratified for group distribution was used.^49^ Results were displayed using a cost‐effectiveness plane and acceptability curve with various willingness to pay (WTP) thresholds (40,000 to 200,000 EUR per QALY gained), demonstrating the probability of cost‐effectiveness.

#### 2.4.4 Additional analyses

We also analyzed the difference between the two study arms over time. *T*‐tests were used to compare outcomes of PlwD in the waiting‐control group at baseline, six months after receiving usual care, and at 12 months after receiving the intervention. Also, subgroup cost‐effectiveness analyses were carried out for PlwD living alone versus those not living alone.

## 3 RESULTS

### 3.1 Sample characteristics

Study enrollment started on January 1, 2021, and ended on December 31, 2022. The follow‐up was completed on August 31, 2023. Three hundred ninety‐one PlwD were included in the efficacy ITT analysis (178 intervention group, 213 usual care). Fifty‐four PlwD passed away between baseline and the 6‐month follow‐up. A total of 358 received the intended treatment (PPA; 165 intervention group, 193 usual care). Three hundred thirty‐two PlwD were included in the cost‐effectiveness analyses. No statistical differences existed between included PlwD and those who dropped out concerning age, sex, and DemTect score (Table S1).

At baseline, PlwD were, on average, 80.6 years old, mostly female (57%), living with a caregiver (59%), and mildly to moderately cognitively impaired (86%) (Table 1). There were no significant differences in PlwD characteristics between the groups, except for the availability of a caregiver (51% vs 38%, *p* = 0.014) and functional impairment (5.5 vs 6.0, *p* = 0.023).

**TABLE 1:** Participant characteristics of the sample (efficacy analysis).

	Total sample (*n* = 391)	Usual care (*n *= 213)	Intervention group (*n* = 178)	*p* value^a^
***Demographics***				
Age, mean (SD)	80.6 (7.0)	81.0 (6.8)	79.9 (7.1)	0.099
Sex (female), *n* (%)	222 (56.8)	122 (57.3)	100 (56.2)	0.767
Caregiver included, *n* (%)	176 (45.0)	108 (50.7)	68 (38.2)	**0.014**
Living alone, *n* (%)	160 (40.9)	89 (41.8)	71 (39.9)	0.757
Nationality (German), *n* (%)	405 (98.1)	221 (98.7)	184 (97.4)	0.478
Education				
Lower secondary school	161 (40.2)	85 (39.7)	76 (40.6)	0.919
Upper secondary school	240 (59.9)	129 (60.3)	111 (59.4)	
Vocational program	292 (73.9)	155 (73.5)	137 (74.5)	0.909
Advanced vocational school	54 (13.5)	33 (15.4)	21 (11.2)	0.243
Non‐university tertiary institution	31 (7.7)	19 (8.9)	12 (6.4)	0.454
University	54 (13.5)	28 (12.9)	26 (14.1)	0.771
***Clinical characteristics***				
Cognitive status (MMSE), mean (SD)	17.7 (7.4)	18.1 (7.2)	17.3 (7.6)	0.292
Mild dementia, *n* (%)	180 (46.0)	99 (46.5)	81 (45.5)	
Moderate dementia, *n* (%)	158 (40.4)	87 (40.9)	71 (39.9)	0.875
Severe dementia, *n* (%)	53 (13.6)	27 (12.7)	26 (14.6)	
Depression (GDS), mean (SD)	3.6 (2.8)	3.7 (2.9)	3.5 (2.6)	0.616
No depression, *n* (%)	334 (85.4)	179 (84.0)	155 (87.1)	0.472
Depression, *n* (%)	57 (14.6)	34 (16.0)	23 (12.9)	0.472
Number of diagnoses^b^, mean (SD)	11.3 (9.6)	12.1 (10.1)	10.3 (8.9)	0.059
Functional impairment (B‐ADL), mean (SD)	5.7 (2.3)	5.5 (2.3)	6.0 (2.2)	**0.023**
Number of drugs taken^c^, mean (SD)	6.7 (3.6)	6.6 (3.5)	6.8 (3.8)	0.599
Mobility impairment (DEMMI), mean (SD)	57.6 (17.1)	56.8 (15.7)	58.6 (18.7)	0.295

Abbreviations: B‐ADL, Bayer Activities of Daily Living, range 0 to 10, lower score indicates better performance; DEMMI, de Morton Mobility Index, range 0 to 100, higher scores indicate a higher level of mobility; GDS, Geriatric Depression Scale, sum score 0 to 15, score ≥ 6 indicates depression; MMSE, Mini‐Mental Status Examination, range 0 to 30, higher score indicates better cognitive function, 30 to 20: mild dementia, 19 to 10: moderate dementia, ≤9: severe dementia; SD, standard deviation.

^a^ Bold numbers indicate a statistically significant difference between groups based on generalized linear (metric variables) or logistic regression models (categorical variables) with random intercepts for the general practitioner (metric variables), representing the cluster.

^b^ Number of ICD‐10 diagnoses recorded in medical record of treating general practitioners.

^c^ Number of regular drugs taken based on cabinet review at patients' homes.

### 3.2 Primary and secondary outcomes

The ITT analyses showed a significant decrease in the primary outcomes number of unmet needs after six months for CANE (*b* = 0.26 [95% CI: 0.17 to 0.40], *p* < 0.001) in the intervention group. This means that the number of unmet needs after six months in the intervention group (PlwD receiving dementia care management by nurses with extended roles) was 74% lower than in the control group. The results of the PPA confirmed these effects for the primary outcome. For CANE, the observed intervention effect was even higher than in the ITT analysis (*b* = 0.20 [95% CI: 0.13 to 0.33], *p* < 0.001). In the PPA, the number of unmet needs was 80% lower than in the control group. A subgroup analysis across all CANE dimensions is demonstrated in Table S2.

For the secondary outcome quality of life, the ITT analysis demonstrated that PlwD receiving dementia care management by nurses with extended roles had a significantly higher HRQoL (AMD 0.04 [95% CI: 0.01 to 0.07], *p* = 0.017) compared to usual care. A positive tendency toward a lower caregiver burden after receiving the intervention (Table 2) was not statistically significant (AMD −1.22 [95% CI, −4.27 to 1.84]; *p* = 0.434) after 6 months. The difference between six and 12 months (i.e., patients of the waiting‐control group who received the intervention after six months of usual care and those who received the intervention after baseline), which was not the primary emphasis in this analysis, shows a slightly larger effect in reducing caregiver burden (AMD −2.05 [95%CI, −0.12 to 4.22]; *p* = 0.064). There was also no effect on HRQoL measured by QoL‐AD (AMD 0.03 [95% CI: −0.03 to 0.08]; *p* = 0.347). A description of outcomes, mean group differences at baseline and after 6 months (and also after 12 months for the waiting‐control group), and effect sizes are demonstrated in Tables 2 and 3.

**TABLE 2:** Description of treatment effect on patient and caregiver outcomes.

	Intervention Mean (SD)	Usual care Mean (SD)	Group differences Mean (SE)	Effect size Cohens‐d [95% CI]	*p* value [95% CI]
**Unmet needs (CANE)**					
Baseline	2.3 (2.5)	2.3 (2.7)	−0.07(0.27)		0.788 [−0.60, 0.45]
6‐month follow‐up	0.5 (1.2)	1.5 (2.4)	1.02 (0.20)	0.55 [0.32, 0.73]	**0.001** [0.63, 1.40]
Difference between 0 and 6 months	−1.8 (2.3)	−0.8 (2.5)	–		–
12‐month follow‐up (waiting group)	–	0.2 (0.6)	–		–
Difference between 6 and 12 months^a^	–	−1.3 (0.2)^a^	–		**0.001** [−1.65, −1.04]^a^
**Caregiver burden (ZARIT)**					
Baseline	24.8 (12.1)	23.6 (13.6)	−1.23 (2.02)		0.542 [−5.22, 2.75]
6‐month follow‐up	24.0 (11.0)	24.2 (12.7)	0.24 (1.87)	0.02 [−0.28, 0.32]	0.896 [−3.44, 3.93]
Difference between 0 and 6 months	−0.8 (9,8)	+0.6 (7.7)	–		–
12‐month follow‐up	–	22.1 (11.8)	–		–
Difference between 6 and 12 months^a^	–	−2.05 (1.09)^b^	–		**0.064 [−0.12, 4.22]**
**Quality of Life (QoL‐AD) Caregiver**					
Baseline	2.5 (0.4)	2.5 (0.4)	−0.00 (0.06)		0.969 [−0.13, 0.12]
6‐month follow‐up	2.4 (0.4)	2.5 (0.4)	0.06 (0.06)	0.16 [−0.14, 0.47]	0.293 [−0.05, 0.17]
Difference between 0 and 6 months	−0.1 (0.3)	0.0 (0.3)	–		–
12‐month follow‐up	–	2.4 (0.3)	–		–
Difference between 6 and 12 months^a^	–	−0.04 (0.02)^b^	–		0.092 [−0.01, 0.09]
**Quality of Life (QoL‐AD) PlwD**					
Baseline	2.7 (0.4)	2.7 (0.4)	0.03 (0.04)		0.377 [−0.04, 0.11]
6‐month follow‐up	2.7 (0.3)	2.7 (0.3)	−0.01 (0.03)	−0.03 [−0.23, 0.17]	0.743 [−0.08, 0.05]
Difference between 0 and 6 months	0.0 (0.3)	0.0 (0.3)	–		–
12‐month follow‐up	–	2.6 (0.3)	–		–
Difference between 6 and 12 months^a^	–	0.03 (0.02)^b^	–		0.152 [−0.01, 0.07]
**Quality of Life (EQ‐5D‐5L) Caregiver**					
Baseline	0.71 (0.20)	0.64 (0.25)	−0.07 (0.04)		**0.044** [−0.14, −0.01]
6‐month follow‐up	0.71 (0.19)	0.71 (0.22)	−0.00 (0.03)	−0.02 [−0.32, 0.28]	0.905 [−0.07, 0.06]
Difference between 0 and 6 months	0.0 (0.1)	+0.08 (0.2)	–		–
12‐month follow‐up	–	0.68 (0.22)	–		–
Difference between 6 and 12 months^a^	–	−0.03 (0.02)^b^	–		**0.066 [−0.00, 0.07]**
**Quality of Life (EQ‐5D‐5L) PlwD**					
Baseline	0.76 (0.20)	0.74 (0.24)	−0.02 (0.02)		0.286 [−0.07, 0.02]
6‐month follow‐up	0.80 (0.17)	0.75 (0.21)	−0.05 (0.02)	−0.26 [−0.46, −0.06]	**0.009** [−0.09, −0.01]
Difference between 0 and 6 months	+0.04 0.0 (0.2)	+0.01 (0.2)	–		–
12‐month follow‐up	–	0.75 (0.21)	–		–
Difference between 6 and 12 months^a^	–	−0.01 (0.01)^a^	–		0.533 [−0.02, 0.03]

*Note*: Bold numbers indicate (at least half‐sided) statistically significant difference between groups; for statistical comparison between groups *t*‐tests were calculated.

Abbreviations: CANE, Camberwell Assessment of Need for the Elderly; CI, confidence interval; EQ‐5D‐5L, The EuroQol 5‐Dimension 5‐level questionnaire; PlwD, people living with dementia; QoL‐AD, Quality of Life in Alzheimer's Disease; SD, standard deviation; SE, standard error; ZARIT, Zarit Caregiver Burden Interview scale.

^a^ Difference between patients of the waiting‐control group that received the intervention after 6‐month care as usual (FU2) and those who received the intervention after baseline (intervention group, FU1); here difference between FU1 and FU2, *n* = 203.

^b^ *n* = 101.

**TABLE 3:** Poisson regression analyses for treatment effect of intervention compared with usual care for primary outcome (number of unmet needs, CANE) and secondary outcomes (caregiver burden and quality of life) after 6 months of intervention.

	ITT (*n* = 391)^a^		PP (*n* = 301)^a^	
Primary outcome Unmet needs (CANE)	(Back‐transformed^b^) coefficient *b* (95% CI)	*p* value	(Back‐transformed^b^) coefficient *b* (95% CI)	*p* value
Study group (ref: control group)	0.26 (0.17 to 0.40)	<0.001^**^	0.20 (0.13 to 0.33)	<0.001^**^
Gender (ref. male)	1.00 (0.71 to 1.40)	0.983	1.18 (0.88 to 1.58)	0.276
Living status (ref. not living alone)	1.36 (0.96 to 1.92)	0.084	1.09 (0.81 to 1.47)	0.582
Age	0.99 (0.97 to 1.02)	0.592	0.99 (0.97 to 1.01)	0.301
Baseline unmet needs	1.15 (1.11 to 1.19)	<0.001^**^	1.15 (1.10 to 1.20)	<0.001^**^

	Adjusted Mean		
Secondary outcomes	Intervention Mean (SE) [CI]	Waiting control group Mean (SE) [CI]	Difference Mean (SE) [CI]
**Caregiver burden (ZARIT)**			
(*n* = 176)	23.04 (1.21) [20.67, 25.41]	24.26 (0.98) [22.34, 26.17]	−1.22 (1.56) [−4.27, to 1.84]
**Quality of life (QoL‐AD) PlwD**			
(*n* = 391)	2.69 (0.02) [2.64, 2.73]	2.66 (0.02) [2.62, 2.70]	0.03 (0.03) [−0.03, 0.08]
**Quality of life (EQ‐5D‐5L) PlwD**			
(*n* = 391)	0.797 (0.01) [0.774, 0.820]	0.758 (0.01) [0.737, 0.780]	**0.04 (0.02) [0.01, 0.07]** ^*^

Abbreviations: CI, confidence interval; ITT, intention to treat; PP, per protocol; PlwD, people living with dementia; SE, standard error.

Interpretation: In contrast to linear regression models, in Poisson regression and negative binomial regression models, the dependent variable is not modeled directly. Instead, the logarithm of the variable is modeled, so the regression coefficients have to be back‐transformed using an exponential function. As a consequence, the back‐transformed coefficient cannot be interpreted as in linear regression models. A coefficient of 1 equals no difference in treatment effects. Values between 0 and 1 indicate a lower dependent variable for the intervention group compared to the control group, whereas values greater than 1 can be interpreted as a greater value for the dependent variable compared to the control group. In addition, a significant treatment effect for these non‐linear regression models is defined as a 95% confidence interval not containing the value 1.

^a^ Mixed‐effects model with random effects for medical practices and networks (both intercept only); predictor of interest was the study group.

^b^ Back‐transformation of coefficients was performed using exponential function for better interpretation.

* *p* < 0.05.

** *p* < 0.001; linear regression analyses adjusted for age, sex, living situation and baseline value; the study group was the predictor of interest; p‐values are given two‐sided.

### 3.3 Cost‐effectiveness outcomes

Total costs after six months were 5,107 EUR (95% CI: 4,550 to 5,664) for the intervention and 3,682 EUR (95% CI: 3,129 to 4,236) for the usual care group. The intervention was associated with significantly increased costs for medical treatments, especially for therapies, but led to lower costs in other medical cost categories, especially for neurologist and psychologist treatments and other specialist visits. There was no effect on institutionalization costs and costs for formal care. Regarding informal care, the intervention group showed significantly lower costs for support per month for ADL/IADL provided by the informal caregiver.

From a payer perspective, the intervention group had significantly higher QALYs (0.01, 95% CI: −0.001 to 0.018) with significantly higher costs (1,425 EUR, 95% CI: 638 to 2,211), resulting in an ICER of 142,500 EUR per QALY gained. Table 4 and Tables S3 and S4 summarize the mean and adjusted costs and QALY results.

**TABLE 4:** Unadjusted and adjusted mean cost and effects, and incremental cost‐effectiveness ratio (payer perspective and societal perspective).

	Unadjusted mean			Adjusted mean		
Payer perspective (total sample, n = 332)	Intervention (*n* = 165) Mean (SE) [95% CI]	Usual care (*n* = 167) Mean (SE) [95% CI]	Difference Mean (SE) [95% CI]	Intervention (*n* = 165) Mean (SE) [95% CI]	Usual care (*n* = 167) Mean (SE) [95% CI]	Difference Mean (SE) [95% CI]
**Medical treatment**	**3,022 (229)** **[2,570, 3,474]**	**2,372 (194)** **[1,989, 2,756]**	**650 (300)** **[60, 1,240]** *	**2,991 (210)** **[2,577, 3,405]**	**2,403 (209)** **[1,992, 2,814]**	**588 (297)** **[4, 1,172]** *
GP treatments	77 (4) [70, 84]	76 (4) [69, 83]	1 (5) [−10, 10]	77 (4) [69, 84]	76 (4) [69, 83]	1 (5) [−10, 10]
Neurologist and psychologist treatments	67 (16) [35, 99]	70 (19) [32, 108]	−3 (25) [−53, 47]	66 (18) [30, 101]	71 (18) [36, 106]	−6 (25) [−55, 44]
Other specialists^a^	131 (11) [109, 153]	148 (14) [121, 176]	−17 (18) [−53, 18]	129 (13) [104, 155]	150 (13) [13, 175]	−21 (18) [−56, 15]
Therapies^b^	764 (85) [596, 931]	382 (51) [282, 482]	381 (98) [188, 575] ***	750 (68) [616, 884]	396 (68) [262, 529]	354 (96) [165, 544] ***
In‐hospital treatments^c^	584 (156) [277, 892]	446 (135) [180, 712]	138 (206) [−266, 543]	574 (146) [286, 861]	457 (145) [171, 742]	117 (206) [−289, 523]
Medications	913 (78) [759, 1,067]	882 (84) [716, 1,048]	31 (115) [−195, 256]	913 (81) [753, 1,072]	883 (81) [724, 1,041]	31 (115) [−195, 256]
Medical aids	487 (64) [360, 613]	367 (55) [259, 475]	119 (84) [−46, 285]	483 (60) [366, 601]	371 (59) [254, 488]	113 (84) [−53, 279]
**Formal care**	**1,579 (150)** **[1,283, 1,875]**	**1,296 (1,911)** **[1,004, 1,588]**	**283 (211)** **[−131, 697]**	**1,596 (149)** **[1,304, 1,889]**	**1,279 (148)** **[989, 1,570]**	**317 (210)** **[−96, 730]**
Ambulatory care^d^	1,280 (114) [1,055, 1,506]	1,051 (1,396) [838, 1,264]	229 (157) [−79, 538]	1,296 (108) [1,083, 1,509]	1,035 (108) [824, 1,247]	261 (153) [−40, 562]
Nursing home	299 (83) [135, 463]	245 (101) [45, 445]	53 (131) [−204, 311]	300 (93) [118, 482]	244 (92) [63, 425]	56 (131) [−201, 313]
**Total healthcare cost**	**4,601 (305)** **[4,000, 5,203]**	**3,669 (259)** **[3,157, 4,181]**	**933 (400)** **[146, 1,719]** *	**4,587 (283)** **[283, 5,144]**	**3,682 (281)** **[3,129, 4,236]**	**905 (400)** **[118, 1,691]** *
**Cost for intervention**	520 (0)	0 (0)	520 (0)	520 (0)	0 (0)	520 (0)
**Total costs (including intervention costs)**	**5,121 (305)** **[4,520, 5,723]**	**3,669 (259)** **[3,157, 4,181]**	**1453 (400)** **[666, 2,239]** ***	**5,107 (283)** **[4,550, 5,664]**	**3,682 (281)** **[3,129, 4,236]**	**1,425 (400)** **[638, 2,211]** ***
QALYs	0.393 (0.01) [0.381, 0.406]	0.374 (0.01) [0.358, 0.390]	0.02 (0.01) [−0.001, 0.040]	0.388 (0.01) [0.382, 0.394]	0.379 (0.01) [0.373, 0.386]	0.01 (0.01) [−0.001, 0.018]

	Incremental cost per QALY gained		72,650€/ QALY	Incremental cost per QALY gained		142,500€/ QALY
**Societal perspective** **(subsample: patients with informal caregiver, *n* = 139)**	**Intervention (*n* = 61)** Mean (SE) [95% CI]	**Usual care (*n* = 78)** Mean (SE) [95% CI]	**Difference** Mean (SE) [95% CI]	**Intervention (*n* = 61)** Mean (SE) [95% CI]	**Usual care (*n* = 78)** Mean (SE) [95% CI]	**Difference** Mean (SE) [95% CI]
**Total healthcare cost (payer perspective)**	**3754 (415)** **[2924, 4584]**	**2990 (296)** **[2400, 3581]**	**764 (497)** **[−219, 1747]**	**3770 (375)** **[3028, 4513]**	**2978 (331)** **[2323, 3633]**	**793 (505)** **[−206, 1792]**
**Informal care**	**15,645 (1453)** **[12,739, 18,551]**	**21,852 (1480)** **[18,904, 24,800]**	**−6207 (2110)** **[−10,380, −2034]** **	**15,280 (1557)** **[12,200, 18,361]**	**22,138 (1374)** **[19,420, 24,856]**	**−6857 (2096)** **[−11,003, −2712]** ***
Support for ADL/IADL provided by caregiver	13,321 (1457) [10,408, 16,235]	20,134 (1510) [17,127, 23,140]	−6812 (2139) [−11,042, −2582] **	12,916 (1569) [9813, 16,019]	20,451 (13,84) [17,713, 23,189]	−7535 (2111) [−11,711, −3359] ***
Support ADL/IADL provided by others	2324 (189) 1946, 2702	1719 (184) [1352, 2085]	605 (267) [77, 1133] *	2365 (200) [1969, 2760]	1687 (176) [1338, 2035]	678 (268) [146, 1209] *
**Total healthcare cost**	**19,399 (1459)** **[16,480, 22,319]**	**24,842 (1488)** **[21,880, 27,805]**	**−5443 (2121)** **[−9637, −1250]** *	**19,050 (1578)** **[15,930, 22,171]**	**25,115 (1392)** **[22,362, 27,868]**	**−6065 (2123)** **[−10,264, −1866]** **
**Cost for intervention**	520 (0)	0 (0)	520 (0)	520 (0)	0 (0)	520 (0)
**Total costs (including intervention costs)**	**19,919 (1459)** **[17,000, 22,839]**	**24,842 (1488)** **[21,880, 27,805]**	**−4923 (2121)** **[−9117, −730]** *	**19,570 (1578)** **[16,450, 22,691]**	**25,115 (1392)** **[22,362, 27,868]**	**−5545 (2123)** **[−9744, −1346]** **
**QALYs**	**0.388 (0.01)** **[0.363, 0.413]**	**0.385 (0.01)** **[0.361, 0.408]**	**0.00 (0.02)** **[−0.031, 0.037]**	**0.385 (0.01)** **[0.375, 0.395]**	**0.387 (0.01)** **[0.378, 0.396]**	**0.00 (0.01)** **[−0.016, 0.012]**
	**Incremental cost per QALY gained**		**Intervention dominates**	**Incremental cost per QALY gained**		**Intervention dominates**

Abbreviations: ADL, Activities of Daily Living; CI, confidence interval; GP, general practitioner; IADL, instrumental Activities of Daily Living; QALY, Quality‐adjusted life year; SE, standard error.

^a^ Include internist, gynecologist, surgeon, orthopedist, dermatologist, ophthalmologist, urologist, and dentist.

^b^ Include physiotherapy, occupational therapy, speech therapy, and medical foot care.

^c^ Include rehabilitation, and hospital stays.

^d^ Include household assistants and outpatient nurses.

* p‐value ≤0.05, ** p‐value ≤0.01, ***p‐value ≤0.001, for statistical comparison between groups t‐tests (unadjusted mean) and multivariate regression model adjusted for age, gender, living situation were calculated.

**FIGURE 2 alz70727-fig-0002:**
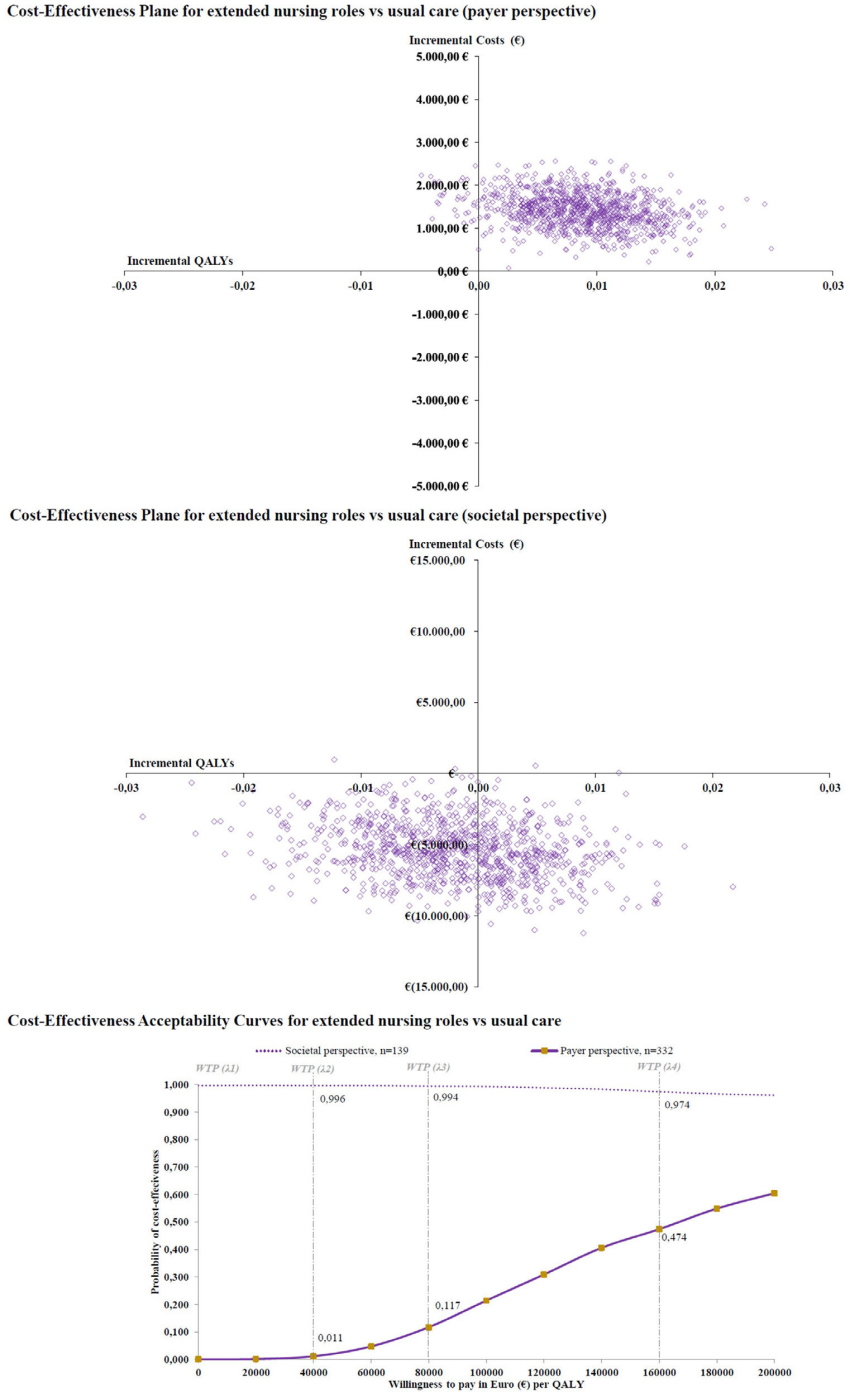
Cost‐effectiveness plane and acceptability curves for extended nursing roles versus usual care.

The probability of the intervention being cost‐effective was 47% at a WTP threshold of 160,000 EUR per QALY gained, as represented in Figure 2.

From a societal perspective that includes informal care and QALYs of caregivers, there was no gain in QALY (0.00, 95% CI: −0.016 to 0.012) but a significant decrease in costs (−5,545 EUR, 95% CI: −9,744, −1,346) mainly driven by lower utilization of informal care provided by caregivers, indicating that the intervention dominated usual care. The probability of cost‐effectiveness was 97% at a WTP threshold of 160,000 EUR per QALY gained (Figure 2).

Sensitivity analyses for PlwD living alone versus those not living alone demonstrated that the intervention was more likely to be cost‐effective in PlwD living alone (Figure S1). The gain in QALY (living alone: *b* = 0.02, 95% CI: 0.007 to 0.036, *p* = 0.004; not alone: *b* = 0.00, 95% CI: −0.012 to 0.010, *p* = 0.857), incremental costs (living alone: b = 892, 95% CI: −255 to 2,040, *p* = 0.126; not alone: *b *= 1,734, 95% CI: 660 to 2,808, *p* = 0.002), and the probability of cost‐effectiveness was better in PlwD living alone (living alone: 97.3% at WTP 160,000 EUR/QALY; not alone: 5.7%).

## 4 DISCUSSION

Extended nursing roles significantly reduce unmet healthcare needs (by 74%) and improve PlwD's HRQoL and QALY after six months. There was a positive tendency toward a reduction of caregiver burden (not statistically significant). Extended nursing roles were likely to be cost‐effective after six months. Sensitivity analyses confirmed these results and revealed that extended nursing roles were primarily cost‐effective in those living alone.

Evidence concerning the effect of innovative care models on unmet needs reduction is rare.^28^ However, RCTs^50^, ^51^ indicated that a home‐based care coordination intervention for elders with memory disorders and a liaison intervention for PlwD in residential care, respectively, significantly reduced unmet needs. Our results align with these findings, demonstrating a significant reduction in unmet needs of 76% in the intervention group compared with the usual care controls. Notably, there was also a significant reduction in unmet needs in the controls. Thus, the increased awareness through conversation with the treating general practitioner about the study and the unmet needs assessment conducted by the study nurses already had an impact. After six months, this effect was significantly lower than that observed in the intervention group. The effect of the intervention may be larger in a real‐world usual care scenario in which PlwD and caregivers are not exposed to the study elements.^50^

Another goal of extended nursing roles within home support programs is to reduce caregiver burden. In a meta‐analysis, four studies evaluated the impact of case management approaches to home support PlwD on caregiver burden over six months, yielding uncertain effects,^14^ which aligns with our results. While there was no significant effect on caregiver burden after six months in our study, we found a lower caregiver burden after 12 months in the waiting‐control group who received the intervention between months seven and 12. The absence of an effect after six months in some of the previous studies and our study could be attributable to the short intervention and observation periods. Michalowsky et al.^52^ recently demonstrated a significant reduction in caregiver burden 36 months after a dementia care management intervention.

Some studies reported better HRQoL of PlwD receiving collaborative nurse‐led care management over 12 and 18 months,^52^, ^53^, ^54^ while others did not.^17^, ^55^ Reilly et al. reports three studies^53^, ^55^, ^56^ assessing HRQoL of participants applying various scales with no significant effects on participants' HRQoL at 4, 6, 12, and 18 months.^14^ Our results align with these inconsistent findings. While we found no effect on overall HRQol with the QoL‐AD instrument, the EQ‐5D‐5L showed significant improvement in HRQoL after 6 months in the intervention group, highlighting the challenge of measuring HRQoL in dementia diseases.^57^ Neither generic nor specific measures assess every aspect of quality of life. Aguierre et al.^58^ showed higher reliability scores and better performance of the EQ‐5D compared to dementia‐specific measures like QoL‐AD for mild and moderate levels of cognitive impairment.^58^ Notably, the observed change in EQ‐5D‐5L scores exceeded the MID of 0.05 reported for non‐surgical interventions, suggesting that the improvement in HRQoL is likely to be clinically meaningful. Further interventional studies should evaluate the sensitivity of various measures to detect HRQoL changes in PlwD. Cost‐effectiveness results were inconclusive.^59^, ^60^, ^61^ The systematic review of Pimouguet et al.^62^ included three studies of collaborative care programs without any evidence for savings.^63^, ^64^, ^65^ The meta‐analysis of Reilly et al.^14^ indicated that costs may be reduced in the first and second year.^66^, ^67^, ^68^ Michalowsky et al.^52^, ^61^ confirmed this, indicating that collaborative dementia care management approaches will likely be cost‐effective over 24 months. Cost‐effectiveness considering subsequent long‐term effects, such as reduced hospitalization or delayed institutionalization, is lacking. Our results revealed an adjusted ICER of 142,500 EUR/QALY already after six months, indicating that extending nursing roles is very likely a cost‐effective strategy with respect to current WTP thresholds of high‐income countries.^69^ Noticeably, our results indicated lower costs for neurologist and psychologist treatments and other specialist treatments, underlining the support and relief of physicians and other specialists by extending nursing roles in dementia care.

Previous studies have shown that PlwD living alone benefit more from collaborative dementia care management models.^52^, ^61^, ^70^ The InDePendent study confirmed this, with sensitivity analyses demonstrating greater QALY gains at only slightly increased costs, resulting in incremental cost‐effectiveness ratios well below typical willingness‐to‐pay thresholds. These differences likely reflect varying levels of informal support: PlwD living alone often lack caregivers to identify and address their unmet needs, whereas those living with others usually receive informal support that reduces their additional benefit from an intervention. Consequently, implementing dementia care management with extended nursing roles may be particularly advantageous for individuals living alone.[Fig alz70727-fig-0002]

The pronounced benefit for this subgroup aligns with existing literature highlighting their increased vulnerability to social isolation, unmet care needs, and poorer health outcomes.^71^ This underscores the critical need for targeted, holistic interventions that leverage advanced nursing skills to provide patient‐centered care. From a policy perspective, allocating resources to programs addressing the unique needs of individuals living alone may improve quality of life and reduce healthcare costs. These findings emphasize the importance of stratifying intervention effects by living situation. Further research is warranted to identify factors driving these differences and to refine dementia care strategies accordingly.

Our analysis indicates no substantial selection bias due to drop‐out, supporting the robustness of the primary findings. The multivariate drop‐out analysis (Table S1) revealed no statistically significant predictors for study attrition after baseline assessment. Neither group allocation nor sociodemographic factors such as sex, age, living situation, or caregiver availability were significantly associated with loss to follow‐up. A non‐significant trend was observed for lower unmet needs and male sex, suggesting that individuals with fewer needs and male participants may have been slightly more likely to drop out. Overall, the analysis indicates no substantial selection bias due to drop‐out, supporting the robustness of the primary findings.

The findings provide important implications for health policy, especially in the context of addressing demographic change and workforce shortages in primary care. The demonstrated efficacy and cost‐effectiveness of extended nursing roles in dementia care offer a strong rationale for expanding nursing scopes of practice. Nurses with advanced training can effectively contribute to interprofessional care by addressing unmet needs, improving HRQoL, and supporting physicians through task‐sharing models. Policymakers should consider creating regulatory frameworks and reimbursement structures that support the sustainable integration of such roles into routine care. Scaling up evidence‐based nursing interventions, as tested in this study, may significantly strengthen primary care capacity and improve access to personalized dementia care across diverse healthcare settings.

In clinical practice, the results support the implementation of nurse‐led dementia care models as a complementary component of primary care services. Specially trained nurses can provide continuous, individualized support through home visits, needs assessments, coordination of services, and targeted interventions – especially for people living alone. To realize the full potential of such models, practical steps include investing in academic training programs for advanced nursing roles and developing interdisciplinary care pathways. These findings can thus inform care organizations, providers, and educators seeking to implement advanced dementia care in community‐based settings.

### 4.1 Limitations

The primary outcome was measured using the CANE. While CANE is sensitive to identifying unmet needs in community‐dwelling PlwD,^72^ it may not consider all relevant need domains and is not specific enough within the existing needs areas, represented by a marginal average number of unmet needs that can be detected.^73^ Hence, there is a need for a more comprehensive, IT‐based needs assessments for PlwD. Furthermore, the results are based on a 6‐month intervention. Effects would probably be higher after longer periods of observation. In routine care practice, healthcare services are carried out continually rather than over a defined period, as in this study. This could potentially yield higher effects, which should be considered in future evaluations. The validity of the assessed data might be limited in terms of completeness and accuracy due to PlwD's cognitive limitations. Additionally, data assessment commenced in January 2021, prior to the last COVID‐19‐related lockdown in April 2021. Physician consultations, hospital admissions, care service visits, and medication prescriptions were reduced during this time,^74^ which may have affected the intervention. However, the study was completed in August 2023. Therefore, this bias might be small. Also, identification of PlwD was based on a DemTect procedure,^20^ a widely used screening tool in primary care and not on a state‐of‐the‐art dementia diagnostic procedure. This could have led to false positive inclusions. However, if the false positives had been evenly distributed across both study groups, this would rather have led to an underestimation of the effect. Finally, the generalizability of our results is limited to mainly mildly cognitively impaired patients within the German healthcare system.

## 5 CONCLUSION

This is the first study evaluating the efficacy and cost‐effectiveness of extended nursing roles for PlwD. We demonstrate positive effects on unmet needs and HRQoL by slightly higher costs. Our results confirmed that extended nursing roles have a high potential to improve the situation of PlwD, health professionals, especially practitioners, and the healthcare system. Addressing unmet needs is currently prioritized in treatment and care of PlwD since no curative therapies are available, even though antibody drugs are now entering the market. Further implementation studies with larger samples, extended intervention periods, longer observational periods, and varying settings are needed to monitor the translation.

## CONFLICT OF INTEREST STATEMENT

The authors declare no potential conflicts of interest, including specific financial interests and relationships and affiliations relevant to the subject of the submitted work. Author disclosures are available in the Supporting Information.

## CONSENT STATEMENT

The local ethics committee at the University Medicine Greifswald approved the study (BB144/20). In addition, secondary approvals were obtained from the local ethics committees at the State Chamber of Physicians of Brandenburg (AS 81(bB)/2020) and Hesse (2020‐2081‐zvBO) (Landesärztekammern). All participants provided their written informed consent before participating in the study. The capacities of people living with dementia (PlwD) to provide informed consent were partially restricted. Therefore, we worked in close collaboration with the GPs, who determined their patients’ capacities to provide valid written informed consent. If a PlwD was not able to provide informed consent, the relative or the legal representative (if available) was invited to provide written consent on the PlwD's behalf. Participants (and caregivers) received a copy of their consent. Study participants could withdraw their consent at any time without negative consequences.

## Supporting information



Supporting Information

Supporting Information

Supporting Information

Supporting Information

Supporting Information

Supporting Information
