# Supplementary figures and images for: Efficacy and cost‐effectiveness of extended nursing roles in dementia care: Results of the cluster‐randomized trial InDePendent
Source: Alzheimers Dement. 2025 Oct 27;21(10):e70727. doi: 10.1002/alz.70727 (PMC12556587; doi:10.1002/alz.70727)

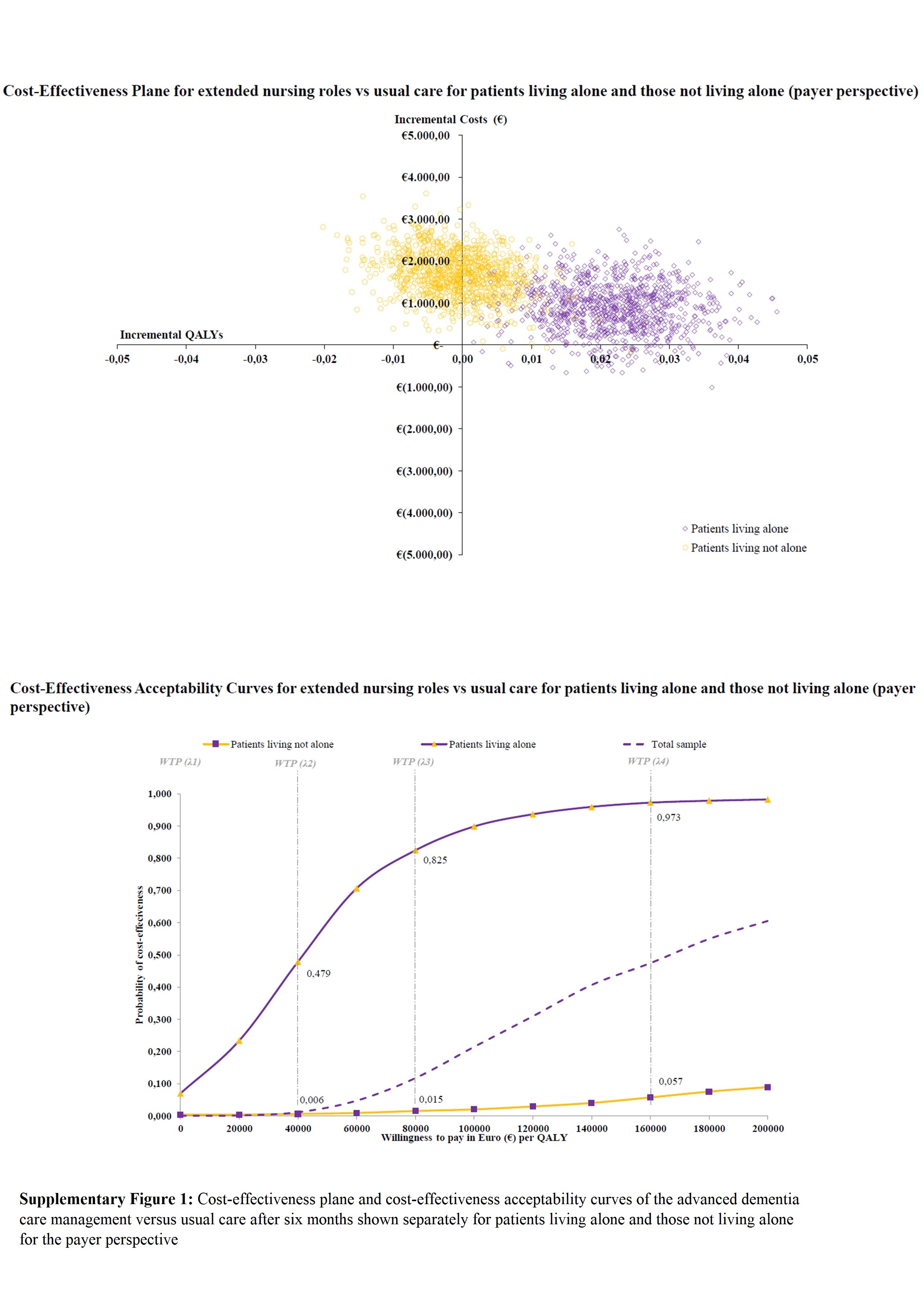

Supplement: Supplementary file 1 — Supporting Information [file ALZ-21-e70727-s002.jpg]
