# Supplementary material for: Efficacy and cost‐effectiveness of extended nursing roles in dementia care: Results of the cluster‐randomized trial InDePendent
Source: Alzheimers Dement. 2025 Oct 27;21(10):e70727. doi: 10.1002/alz.70727 (PMC12556587; doi:10.1002/alz.70727)
Supplement: Supplementary file 2 — Supporting Information [file ALZ-21-e70727-s003.docx]

**Supplementary Table 1:** Drop-out-analysis for patients who dropped out after completing the baseline assessment (n=417)

|  | **Excluded** | |
| --- | --- | --- |
|  | OR (95%-CI) | *p-value* |
| **Lost to follow – up 1** |  |  |
| Study group (Ref. intervention) | 0.91 (0.32 – 2.61) | 0.866 |
| Unmet needs (CANE) | 0.88 (0.75 – 1.02) | 0.092 |
| Sex (Ref. female) | 2.10 (0.90 – 4.89) | 0.086 |
| Informal caregiver (Ref. available) | 1.55 (0.54 – 4.41) | 0.413 |
| Living situation (Ref. alone) | 1.45 (0.50 – 4.19) | 0.492 |
| Age | 0.94 (0.87 – 1.02) | 0.159 |

**Abbreviations:** OR, odds ratios; Ref, reference; CI, confidence interval; SD, standard deviation.

**Footnotes:** Drop out due to death included in cost-utility-analysis as zero cost and utility values; Multivariate logistic regression analysis with random effects for the general practitioner.
