# Supplementary material for: Efficacy and cost‐effectiveness of extended nursing roles in dementia care: Results of the cluster‐randomized trial InDePendent
Source: Alzheimers Dement. 2025 Oct 27;21(10):e70727. doi: 10.1002/alz.70727 (PMC12556587; doi:10.1002/alz.70727)
Supplement: Supplementary file 3 — Supporting Information [file ALZ-21-e70727-s004.docx]

**Supplementary Table 2:** Change of unmet needs over time

|  | **Baseline** | |  | **Follow-Up** | |  | **Relative change** | |
| --- | --- | --- | --- | --- | --- | --- | --- | --- |
| **Unmet need domain (CANE)** | **Intervention** | **Usual care** | **Difference^1^** | **Intervention** | **Usual care** | **Difference^1^** | **Intervention** | **Usual care** |
| Accommodation/housing, n (%) | 3 (1.7) | 4 (1.9) | 0.598 | 0 (0) | 2 (0.9) | 0.503 | -100% | -50% |
| Household tasks, n (%) | 2 (1.2) | 11 (5.2) | **0.023** | 0 (0) | 3 (1.4) | 0.254 | -100% | -73% |
| Food and nutrition, n (%) | 2 (1.1) | 8 (3.8) | 0.119 | 0 (0) | 3 (1.4) | 0.254 | -100% | -63% |
| Personal care, n (%) | 5 (2.8) | 7 (3.3) | 1.000 | 1 (0.6) | 4 (1.8) | 0.382 | -80% | -43% |
| Other practical tasks, n (%) | 3 (1.7) | 0 (0.0) | 0.093 | 0 (0) | 2 (0.9) | 0.503 | -100% | +100% |
| Daytime activities / use of time, n (%) | 15 (8.4) | 21 (9.8) | 0.726 | 4 (2.2) | 17 (8.0) | **0.013** | -73% | -19% |
| Memory or concentration, n (%) | 17 (9.6) | 23 (10.8) | 0.739 | 7 (3.9) | 21 (9.8) | **0.029** | -59% | -9% |
| Seeing or hearing, n (%) | 9 (5.1) | 20 (9.4) | 0.122 | 2 (1.1) | 8 (3.8) | 0.119 | -78% | -60% |
| Mobility, n (%) | 6 (3.3) | 8 (3.7) | 1.000 | 2 (1.1) | 10 (4.7) | 0.073 | -67% | +25% |
| Falls prevention or management, n (%) | 5 (2.8) | 8 (3.8) | 0.779 | 0 (0) | 7 (3.3) | **0.017** | -100% | -13% |
| Continence (bladder/bowel), n (%) | 5 (2.8) | 10 (4.7) | 0.431 | 0 (0) | 9 (4.2) | **0.005** | -100% | -10% |
| Physical health problems, n (%) | 2 (1.1) | 10 (4.7) | 0.073 | 0 (0) | 2 (0.9) | 0.503 | -100% | -80% |
| Taking medication, n (%) | 3 (1.2) | 2 (1.0) | 0.663 | 0 (0) | 2 (0.9) | 0.503 | -100% | 0% |
| Psychotic symptoms, n (%) | 2 (1.1) | 6 (2.8) | 0.300 | 1 (0.5) | 1 (0.5) | 1.000 | -50% | -83% |
| Psychological distress (e.g., anxiety, depression), n (%) | 5 (2.8) | 20 (9.4) | 0.011 | 1 (0.5) | 6 (2.8) | 0.132 | -80% | -70% |
| Information about condition and treatment, n (%) | 7 (3.9) | 13 (6.1) | 0.366 | 0 (0) | 1 (0.5) | 1.000 | -100% | -92% |
| Self-harm or suicidal thoughts, n (%) | 0 (0) | 0 (0) | 1.000 | 0 (0) | 1 (0.5) | 1.000 | — | +100% |
| Unintentional self-harm (e.g., neglect, poor judgment), n (%) | 1 (0.6) | 3 (1.4) | 0.629 | 0 (0) | 1 (0.5) | 1.000 | -100% | -67% |
| Protection from abuse or exploitation, n (%) | 0 (0) | 0 (0) | 1.000 | 0 (0) | 0 (0) | 1.000 | — | — |
| Socially inappropriate behavior, n (%) | 7 (3.9) | 11 (5.2) | 0.634 | 3 (1.7) | 4 (1.9) | 1.000 | -57% | -64% |
| Substance misuse, n (%) | 4 (2.25) | 1 (0.5) | 0.182 | 2 (1.1) | 0 (0.0) | 0.207 | -50% | -100% |
| Social relationships, n (%) | 10 (5.6) | 15 (7.0) | 0.679 | 2 (1.1) | 11 (5.2) | **0.043** | -80% | -27% |
| Partner or intimate relationships, n (%) | 0 (0) | 1 (0.5) | 1.000 | 0 (0) | 1 (0.5) | 1.000 | — | 0% |
| Managing finances, n (%) | 0 (0) | 1 (0.5) | 1.000 | 1 (0.5) | 2 (0.9) | 1.000 | +100% | +100% |
| Accessing benefits and entitlements, n (%) | 12 (6.4) | 13 (6.1) | 0.838 | 0 (0) | 6 (2.8) | **0.034** | -100% | -54% |
| General information or advice, n (%) | 15 (8.4) | 21 (9.8) | 0.726 | 0 (0) | 8 (3.8) | **0.009** | -100% | -62% |
| Caregiver strain / family burden, n (%) | 18 (10.1) | 22 (10.3) | 1.000 | 6 (3.4) | 13 (6.1) | 0.244 | -67% | -41% |
| Mean number (SD) of unmet needs according to the CANE | **2.3 (2.5)** | **2.3 (2.7)** | 0.788 | **0.5 (1.2)** | **1.5 (2.4)** | **0.001** | **-78%** | **-34%** |

**Abbreviations:** CANE, Camberwell Assessment of Need for the Elderly

**Footnotes:** ^1^ for statistical comparison between groups Fisher Exact tests were calculated
