# Supplementary material for: Efficacy and cost‐effectiveness of extended nursing roles in dementia care: Results of the cluster‐randomized trial InDePendent
Source: Alzheimers Dement. 2025 Oct 27;21(10):e70727. doi: 10.1002/alz.70727 (PMC12556587; doi:10.1002/alz.70727)
Supplement: Supplementary file 4 — Supporting Information [file ALZ-21-e70727-s001.docx]

**Supplementary Table 3:** Cost categories and unit costs for monetary valuation of medical, formal and informal healthcare resources and services

| Cost categories | Services | Units | Unit costs^†^ | Unit cost & source for monetary valuation |
| --- | --- | --- | --- | --- |
| Medical care |  |  |  |  |
| Physician treatment | GP or specialists | contact | 22.50-87.56€, depending on specialization | Cost per contact; Bock et al.^1^ |
| Therapies | Occupational therapy, speech therapy, physiotherapy and others | contact | 29.07-64.80€ | GKV Spitzenverband 2023^2^ |
| In-hospital | In-hospital treatment, psychiatric wards and rehabilitation | days | 51.75-645.84€ | Bock et al.^1^ |
| Medications | Regularly prescribed drugs (Rx-drugs) | quantity | 250€ | Pharmaceutical Index of the Scientific Institute of the AOK^3^ |
| Medical aids | Aids such as tub-lifts, tub-seats, walking sticks, walkers and others | quantity | 730€ | Barmer Hilfsmittelreport 2023^4^ |
| Formal care |  |  |  |  |
| Ambulatory nurses | Home care and cleaning assistance provided by professionals | hours | Market price, 13.57-17.10€ | Market prices for Mecklenburg Western-Pomerania |
| Nursing home | Long-term care (institutionalization) | days | 44.66-104.75€, depending on care level* | Pflegestatistik 2021^5^ |
| Informal Care |  |  |  |  |
| Support for ADL/IADL provided by caregiver (hours) | Home care provided by the primary caregiver | hours | 19.72€ | Juan Oliva-Moreno et al.^6^ |
| Support for ADL/IADL provided by others (hours) | Home care provided by family, friends or others | hours | 19.72€ | Juan Oliva-Moreno et al.^6^ |

GP, general practitioner; AOK, allgemeine Ortskrankenkasse; ^*^care level one: mild functional impairment, care level two: moderate functional impairment, care level three: severe functional impairment; ^‡^ when drugs, aids or services were unknown or market prices were not available; ^†^ inflation included.

Footnotes:

^1^ Bock JO, Brettschneider C, Seidl H, et al. [Calculation of standardised unit costs from a societal perspective for health economic evaluation]. Gesundheitswesen. Jan 2015;77(1):53-61. doi:10.1055/s-0034-1374621; ^2^ GKV-Spitzenverband: Heilmittel 2023 (<https://www.gkv-spitzenverband.de/krankenversicherung/ambulante_leistungen/heilmittel/heilmittel.jsp>); ^3^ WIdO (Wissenschaftliches Institut der AOK): GKV-Arzneimittelindex 2016 (<http://www.wido.de/amtl_atc-code.html>); ^4^ Barmer: Heil- und Hilfsmittelreport 2023 (<https://www.bifg.de/publikationen/reporte/heil-und-hilfsmittelreport-2023>); ^5^ Statistisches Bundesamt: Pflegestatistik 2021- Pflege im Rahmen der Pflegeversicherung, Deutschlandergebnisse (<https://www.statistischebibliothek.de/mir/receive/DEHeft_mods_00146747>); ^6^ Oliva-Moreno J, Trapero-Bertran M, Peña-Longobardo LM, del Pozo-Rubio R. The valuation of informal care in cost-of-illness studies: a systematic review. Pharmacoeconomics. 2017;35:331-345.
