# Supplementary material for: Efficacy and cost‐effectiveness of extended nursing roles in dementia care: Results of the cluster‐randomized trial InDePendent
Source: Alzheimers Dement. 2025 Oct 27;21(10):e70727. doi: 10.1002/alz.70727 (PMC12556587; doi:10.1002/alz.70727)
Supplement: Supplementary file 5 — Supporting Information [file ALZ-21-e70727-s005.docx]

**Supplementary Table 4:** Unadjusted healthcare resource utilization, cost and quality-adjusted life of the intervention versus usual care for six months

|  | **Unadjusted Mean** | | |
| --- | --- | --- | --- |
| **Total sample, n=332** | **Intervention** Mean (SE) [CI] | **Usual Care** Mean (SE) [CI] | **Difference** Mean (SE) [CI] |
| ***Healthcare resource use, mean (SD)*** |  |  |  |
| **Medical treatments** |  |  |  |
| Physician treatments (visits) | 3.4 (0.16) [3.09 – 3.72] | 3.38 (0.15) [3.07 – 3.69] | 0.01 (0.22)  [-0.42 – 0.45] |
| Neurologist & Psychologist treatments (visits) | 1.12 (0.20) [0.72 – 1.53] | 1.10 (0.23) [0.63 – 1.57] | 0.02 (0.31)  [-0.59 – 0.64] |
| Specialty Doctors treatment (visits) | 2.76 (0.23) [2.29 – 3.23] | 3.07 (0.28) [2.50 – 3.64] | -0.31 (0.37) [-1.05 – 0.42] |
| Therapies (visits) | 18.16 (1.94) [14.33 – 22.00]** | 9.73 (1.21) [7.34 – 12.13]** | 8.43 (2.28) [3.93 – 12.92]** |
| In-hospital treatments (days) | 1.15 (0.29) [0.58 – 1.72] | 0.84 (0.24) [0.36 – 1.32] | 0.30 (0.37) [-0.43 – 1.05] |
| Medications (per drug) | 6.53 (0.27) [5.98 – 7.08] | 6.53 (0.27) [5.99 – 7.08] | -0.01 (0.39) [-0.77 – 0.76] |
| Medical aids (per aid) | 0.66 (0.08) [0.49 – 0.84] | 0.50 (0.07) [0.35 – 0.65] | 0.16 (0.11) [-0.06 – 0.39] |
| **Formal care** |  |  |  |
| Ambulatory care (hours) | 78.21 (6.85) [64.68 – 91.75] | 62.94 (6.41) [50.28 – 75.60] | 15.27 (9.38) [-3.18 – 33.72] |
| Nursing home (days) | 12.21 (2.18) [7.90 – 16.53] | 12.07 (2.70) [6.73 – 17.41] | 0.14 (3.48) [-6.70 – 6.99] |
| ***Subsample: patients with informal caregiver, n=139*** |  |  |  |
| **Informal care** |  |  |  |
| Support for ADL/IADL provided by caregiver (hours) | 103.18 (31.29) [40.67 – 165.68]** | 360.09 (68.35) [223.69 – 496.49]** | -256.91 (76.30) [-407.84 – -105.98]** |
| Support for ADL/IADL provided by others (hours) | 68.81 (8.70) [51.43 – 86.20]* | 62.17 (9.27) [43.66 – 80.68] | 6.64 (12.74) [-18.56 – 31.85] |
| **p-value ≤0.01, **p-value ≤0.001* | | | |
